# Editorial: Guanine nucleotide exchange factors as determinants of cell fate and development

**DOI:** 10.3389/fcell.2025.1629906

**Published:** 2025-05-29

**Authors:** Vegesna Radha, Katsuhiko Tabuchi, Jacek Z. Kubiak

**Affiliations:** ^1^ CSIR - Centre for Cellular and Molecular Biology, Hyderabad, India; ^2^ Department of Neurohealth Innovation, Institute for Biomedical Sciences, Interdisciplinary Cluster for Cutting Edge Research, Matsumoto, Japan; ^3^ Department of Molecular and Cellular Physiology, Shinshu University School of Medicine, Matsumoto, Japan; ^4^ Dynamics and Mechanics of Epithelia Group, National Centre for Scientific Research (CNRS), Faculty of Medicine, Institute of Genetics and Development of Rennes (IGDR), UMR 6290 CNRS/University of Rennes, Rennes, France; ^5^ Laboratory of Molecular Oncology and Innovative Therapies, Military Institute of Medicine—National Research Institute, Warszawa, Poland

**Keywords:** GTPase, guanine nucleotide exchange factor, signaling, differentiation, cell fate, embryonic development

Embryo development requires precise coordination of molecular and cellular processes. Cell proliferation, migration, fate and 3D spatial organization, all essential during development, are controlled by signalling cascades that regulate gene expression, post translational modifications of proteins and cytoskeletal reorganization. GTPases, which are in turn controlled by the actions of Guanine nucleotide exchange factors (GEFs), GTPase activating proteins (GAPs), and Guanosine dissociation inhibitors (GDIs), enable signal dissemination that determines specific response of cells (Cherfil and Zeghouf, 2013; Kloc et al., 2019). The articles in this Research Topic showcase the importance of GEFs in regulating animal development and in etiopathogenesis of disease. This class of proteins functions as adaptors as well as *bona fide* enzymes (Bos et al., 2007; Radha et al., 2011). Properties and function of GEFs have been understood using biochemical assays, structural analysis, interaction assays, manipulation of their expression, and observing phenotypic consequence of their misexpression and genetic variants. While most GEFs act specifically on their effector GTPases, there are some like RAPGEF1 which act promiscuously on more than one class of GTPases (Sprang, 2001; Chiang et al., 2001). Following their spatiotemporal activation is crucial in understanding and manipulating their function. GEFs play an important role during development as well as in maintaining homeostasis of adult tissues through regulating multiple signalling pathways that control tissue differentiation. They respond to external molecular as well as physical cues driving cellular responses that enable cell fate decisions. Over the years, their role in development has been demonstrated across invertebrate and vertebrate species. Dysregulation of GEFs in a disease context and identification of variants associated with multiple disorders underscores the importance of studying their properties, functions and regulation. Simultaneously, it has been important to develop tools and techniques that enable us to understand and manipulate GEF functions *in vivo* within the context of living cells and organisms.

Several assays have been designed to examine global GEF activity as well as their localized activation in cells (Blaise et al., 2022). In addition, it has been possible to manipulate GEF activity spatially and temporally within cells using a variety of inducible vectors with ease of detection and monitor effector functions downstream of specific GEFs. Pal et al.’s article in this Research Topic describes the use of optogenetic tools to activate specific GTPases by targeting their GEFs to distinct sub-cellular locations and to monitor actin cytoskeletal remodelling and cell behaviour. Pharmacological manipulation to activate or inactivate individual GEF activity has been difficult, and most studies have been carried out through genetic manipulation, or overexpression of proteins. These methods have certain drawbacks, as cellular GEF activation is generally transient and precisely localized. The optogenetic tool described here overcomes many limitations, and provides a good handle to study cellular functions by enabling visual tracking.

The morphogenesis of tissues, and maintaining size and shape of adult tissues is dependent on the physical microenvironment of cells including stiffness of the ECM, and forces generated during movements (Alasaadi and Mayor, 2024). Our understanding at the molecular level of how cells respond to physical/mechanical cues which are important as tissues take the required shape during development is still poor. Cell adhesion molecules respond to forces, which are then transmitted through kinases and GEFs. Several years ago, Tamada et al. (2004) showed how cells respond to stretch of the cytoskeleton, by changing RAPGEF1 structure and causing its activation. In the past decade, we have added evidence demonstrating that GEFs play an important role in mechano-transduction. In this Research Topic, Ohashi et al. review the role of Rho family GTPases involved in mechanical stress responses, emphasizing on the Dbl family of GEFs that act on Rho GTPases. They also discuss how these GTPases regulate the actin cytoskeleton in response to mechanical cues. Physical forces play an important role during tumorigenesis, and metastasis, and therefore the understanding of molecular signalling responses becomes extremally important.

Deregulation of expression and activity of many individual GEFs, as well as polymorphisms and mutations in genes coding for these proteins have been shown to be associated with a variety of disorders, and cancers in particular (Barrio-Real and Kazanietz, 2012; Porras et al., 2021; Farago et al., 2020; Oberley et al., 2012). Maintaining their appropriate levels as well as activity in cells has been shown to be important, as both under-expression and overexpression are observed in solid tumours, like that of the breast, lung, liver and neuroblastomas, as well as in hematopoietic malignancies. In this Research Topic, Njei et al. review the importance of GEFs in etiopathogenesis of Colo-rectal cancers (CRCs), and catalogue the various GEFs known up to date to be associated with CRCs. They highlight the role of GEFs in altering cytoskeleton and cross talks with multiple other pathways contributing to metastatic potential of tumours. In addition, they show how certain GEFs act downstream of muscarinic acid receptors to regulate expression of cell cycle regulatory genes. They also discuss possibilities of using GEFs as biomarkers and therapeutic targets.

CNS development is dependent on appropriate proliferation of precursor cells, their migration, myelination, differentiation, and establishment of connectivity. These processes are controlled by signalling responses to growth factors as well as substrate molecules in the microenvironment (Kelley and Paşca, 2022). Several GEFs play a role in these processes, and their deregulation is associated with many neurological disorders (Scala et al., 2021). The research article by Schäfer et al. in this Research Topic describes the requirement of Vav3, a GEF for Rho family GTPases in myelination of neurons by precursor oligodendrocytes. Generating oligo-spheres from precursor cells derived from normal and Vav3 KO mice, they show deregulated migratory behaviour on ECM matrix proteins using time-lapse photography. This study shows that GEF dependent signalling negatively controls migratory behaviour, important for regulated movement and myelination during CNS development.

As our understanding deepens, future studies will need to bridge molecular mechanisms with physiological outcomes *in vivo*, across tissues and developmental stages. We anticipate that interdisciplinary efforts combining structural biology, live-cell imaging, and genetic tools will be essential to reveal how GEFs orchestrate cell behaviour in diverse contexts. By exploring these molecules beyond their established roles, we may uncover new principles of cell fate regulation and identify novel targets for intervention in developmental disorders and beyond. Because of their activation in response to diverse stimuli and their involvement in multiple downstream effector functions, manipulating individual GEF activity therapeutically may be challenging. Therefore, much remains to be investigated and understood. We hope that this Research Topic will stimulate further interest in studying this class of molecules to better understand developmental processes and enable intervention in diseased states.
